# Application of QSAR Method in the Design of Enhanced Antimalarial Derivatives of Azetidine-2-carbonitriles, their Molecular Docking, Drug-likeness, and SwissADME Properties

**DOI:** 10.22037/ijpr.2021.114536.14901

**Published:** 2021

**Authors:** Zakari Ya’u Ibrahim, Adamu Uzairu, Gideon Adamu Shallangwa, Stephen Eyije Abechi

**Affiliations:** *Department of Chemistry, Faculty of Physical Sciences, Ahmadu Bello University, P.M.B 1045, Zaria. Nigeria.*

**Keywords:** QSAR, design, docking, drug-likeness, Azetidine-2-carbonitriles, P. falciparum, SwissADME

## Abstract

The resistance of the P. falciparum strain to some of the antimalarial drugs has been a dominant dilemma facing the treatment of this fetid disease. This necessitates the detection and development of new antimalarial agents targeting the P. falciparum*.* Azetidine-2-carbonitriles reported for its antimalarial activities, could provide an alternative to the customized antimalarial drugs. Leading to the use of quantitative structure-activity relationship (QSAR) studies, which relates the structures of Azetidine-2-carbonitriles with their activities to generate predictive models. The structures were optimized using density functional theory (DFT) DFT/B3LYP/6-31G* basis set to generate their molecular descriptors, where five predictive models were constructed using the generated descriptors. The models were constructed using the genetic function algorithm component of a material studio, where the model with good statistical parameters, high coefficient of determination (R^2^) = 0.9465, cross-validated R^2^ (Q^2^cv) = 0.8981, Q^2 ^_(L4O)_cv = 0.9272, and highest external validated R^2^ (R^2^_pred_) = 0.6915 was selected as the best model. These statistical results show the robustness, excellent power of prediction, and validity of the selected model. The descriptor, SpMax2_Bhp (the maximum absolute eigenvalue of Barysz matrix for n = 2 was weighted by polarizability), was revealed to be the most influential in the model due to its highest mean effect. The descriptor played a role in the design of sixteen (16) theoretical derivatives of Azetidine-2-carbonitriles using compound 25 as the design template by increasing polarizability of the compounds through substitution of the various group with electron deactivating groups (F, I, Cl, SO_3_H, CN, NO_2_, *etc*.) at different position of the template. The designed compounds were docked with Plasmodium falciparum dihydroorotate dehydrogenase (Pf-DHODH), giving compound D9 the highest binding energy. The designed compounds were further screened for their drug-likeness, where they all pass Lipinski’s RO5. All the compounds show good skin permeability coefficient and have low Gastrointestinal absorption while few compounds D1, D2, D3, D14, and D15 inhibiting the CYP1A2.

## Introduction

The genus Plasmodium is the causative agent of a life-threatening infection, malarial, globally established as one of the most challenging health concerns. Malarial is transmitted within humans through a bite of infected anopheles mosquitoes (1). The global malarial index shows about 228 million malarial cases yearly with 405,000 record mortalities, where the most affected are children below the ages of 5 years, constituting 585,000 (67%) of all cases (2).

Human malarial is transmitted by five species of Plasmodium, namely, Plasmodium ovale (P. ovale), Plasmodium falciparum (P. falciparum), Plasmodium vivax (P. vivax), Plasmodium malariae (P. malariae), and Plasmodium knowlesi (P. knowlesi) (3-4). The bulk of the fatalities are caused by P. falciparum, the most severe of all the species (5). P. falciparum altered the surface of red blood cells once present in the human body through interceding parasite proteins (6). The hemoglobin is ramshackle into amino acids and heme by enzymes cysteine and aspartic proteinases (7). The entire amino acid constituents are assembled into parasite proteins; although only a fraction of heme is incorporated into parasite hemoproteins, the parasite enzymes detoxified the remaining heme (8).

Efficacies of several drugs such as chloroquine, quinine, pyrimethamine, progu-anil, artemisinin, atovaquone, and mefloquine, in treating malarial has been explored. However, the resistance of the P. falciparum strain to some of these drugs has been the major problem facing the treatment of the noxious disease (9). Hence, the detection and development of new antimalarial agents targeting P. falciparum become an extremely important task to curb the accelerated escalation of this resistance. In light of this, Azetidine-2-carbonitriles reported possessing antimalarial activities (10) could provide an alternative application to the routine antimalarial drugs. 

The desire to improve drugs with better antimalarial activities leads to the adoption of quantitative structure-activity relationship (QSAR) studies, an essential process in the field of drug invention and improvement due to its time and cost-effectiveness (11). QSAR is an arithmetical relationship between the structural features (biological activities) of drugs with their physicochemical properties (molecular properties). Through this, substit-utions of various groups at various positions can affect the molecular properties of the compound and hence, instrumentals in the design of antimalarial compounds of novel activities against malarial agents. Various QSAR advances are employed in the studies of biological activities of antimalarial compounds as functions of their molecular properties (12-16). This research focuses on applying QSAR techniques in determining the vital structures of Azetidine-2-carbonitriles, responsible for their antimalarial activities, and utilizing the most important molecular properties in designing derivatives of derivatives Azetidine-2-carbonitriles with enhanced activity against P. falciparum. The drug-like and SwissADME studies of the designed derivatives were conducted, followed by their molecular docking to determine their binding site and energy.

## Experimental

*Collection of dataset and optimization*

The dataset consists of thirty-four derivatives of Azetidine-2-carbonitriles, whose chemical structures and biological activities against the Dd2 strain of *P. falciparum* were extracted from PubChem as presented in the literature (10). Their activities, expressed as EC_50_ (μM), were then converted to pEC_50_ by taking the negative logarithm of the EC_50_ (M) as indicated in Table 1. The structures of the compounds were drawn using a ChemDraw Ultra 12, and saved in *cdx format before exporting into the spartan’14 version 1.1.2 software and then optimized using DFT (DFT/B3LYP/6-31G*) in a vacuum, this is done using the initial molecular geometry (17).

*Descriptors calculation*

The thirty-four [34] optimized Spartan 14 structures saved as SDF format were then exported into PaDEL software where about 1,500 molecular descriptors ranging between 0-3D classes of descriptors were calculated (18). 

*Dataset pre-treatment and division*

The dataset descriptors are treated by eliminating constant value descriptors, excessive values of coefficient of correlation, descriptors with less than 0.001 variance values. The treated data set was divided into 27 training compounds (consisting of 80% of the data set) and 7 test compounds (making up the remaining 20%) with the aid of the DatasetDivision1.2 program by employing the Kennard-Stone’s algorithm method (19).

*Selection of variables and model develo-pment*

Material Studio 8.0 software was employed for the development of a model connecting the biological activities of the Azetidine-2-carbonitriles to their molecular structures. The genetic function algorithm (GFA) component of the material studio was elected to carry out the model development. All possible mixtures of molecular descriptors were searched by the algorithm to produce a good model together with the use of a lack of fit function in measuring the fitness of all individual combinations (20). 

*Model Validation*

The models were subjected to both internal and external validations, where both the leave-one-out (LOO) and leave-many-out (LMO) internal validation techniques were employed. The LOO involves casting away a molecule of the training set before developing a model with the remnant data, and the activity of the discarded compound was in turn predicted by the model, and this was performed across other compounds in the training set. The LMO involves a selection of the group of compounds to validate the developed model. The external validation entails predicting the biological activities of some dataset separated from the training set (test set) applying the model. The best predictive models were chosen based on the values of the coefficient of determination (R^2^), cross-validated R^2^ (Q^2^cv), and the external validated R^2^ (R^2^_pred_) (21). The model with the highest test set (*R*^2^_pred_) prediction was picked as the best model. 

*Descriptors variance inflation factor (VIF)*

The multicollinearity of the model descriptors was investigated employing the variance inflation factor (VIF) (22). The rule of thumb for descriptors VIF (Equation 1) values was set for not greater than 10 as an omen of huge multicollinearity between descriptors (23). The VIF is obtainable by utilizing Equation 1.

$\mathrm{VIF}=\frac{1}{1-R_{i}^{2}}$

 (1)

Where R_i_ denotes the coefficient produced by regressing the descriptor x_i_ against the other descriptors x_j_ (j ≠ i).

*Descriptors Mean effect*

The mean effect refers to the measures of the descriptor’s relevancies in a generated model; it reveals the respective contributions of molecular descriptors to the selected model. The sign of the mean effect indicates the variations of its contribution as a function of descriptor values. The mean effect could be estimated with the aid of Equation 2.

$\mathrm{mean Effect}=\frac{\beta_{j}\sum_{i}^{n}D_{j}}{\sum_{i}^{n}(\beta_{j}\sum_{i}^{n}D_{j})}$

 (2)

where β_j_ Conforms with the descriptor j’s coefficient, D_j_ conforms with each value of matrix descriptor in the training set, and m conforms with the tally of model descriptors present, and n stands for the tally of molecules used as training set (24). 

*Model Applicability Domain (AD)*

The model limitations are defined by its biological space, which relates to the structural realm and response capacity. Interpretation of the relevant space of the model was performed using Williams’s plot. Higher leverage compounds with values exceeding the caution leverage (h*) in addition to determining standardized residual values beyond plus or minus three standard deviation units were regarded as an impairment (25).

*Molecular design*

The ligand-based design method was employed to design compounds possessing improved antimalarial activities. Compounds with enhanced activity are usually refined and modeled through this means by linking the empirical activities of the compounds with their molecular structures. Antimalarial derivatives possessing the highest activity will be utilized as a guide (Template) in designing theoretical compounds with elaborating activities.

*Molecular docking studies*

The molecular docking studies between the ligands and their protein target were carried out with the Molegro Virtual Docker (MVD). The 3D structure of the target protein was extracted from Protein Data Bank (PDB) (www.rcsb.org) and saved in PDB file format. The designed derivatives of Azetidine-2-carbonitriles were docked to a high resolution 2.4 Å crystal structure of Plasmodium falciparum dihydroorotate dehydrogenase (Pf-DHODH) (PDB ID: ITV5). 

*Ligand and Protein Preparation*

The ligands and protein structure saved in PDB format may be lost components such as hydrogen atoms, its charges, etc. The ligands and the protein were imported into the MVD, and were prepared using the preparation wizard (capable of fixing the missing hydrogens and charges) of the MVD. The binding pocket for the interaction of the ligands and the protein target was calculated within the in-built cavity algorithm executed by the MVD software. The MVD cavity detects the algorithm and then performs the molecular docking to predict the ligand’s binding mode and the target protein in the form of a scoring function. 

*Docking Protocol Validation*

The docking protocol was validated through the re-docking of the crystallized ligand unto the binding site of the protein receptor. The initial crystalized ligand was superimposed on the docked pose to produce the value of the root mean square deviation (RMSD). The permitted range of the RMSD value within ≤ 2.0Ǻ validates the docking protocols and confirms its usage in docking (26). 

*In‒silico ADME prediction*

An excellent medication is not necessarily the one with the best binding interactions with the target. Hence, the need to evaluate the ligand’s pharmacokinetic properties, ADME (absorption, distribution, metabolism, and excretion), for their activity within the human system. The compounds that are likely to be taken as oral medication, should be fast and absorb completely from the gastrointestinal tract, distribute in the direction of its target, metabolize slowly, and properly dispense harmlessly. Drug failure has been associated with poor ADME properties (27). The SwissADME, an online ADME prediction tool was deployed in the present studies to predict the drug-like and the pharmacokinetic properties of the sixteen [16] designed derivatives of Azetidine-2-carbonitriles. The predictive absorption for molar refractivity (MR), skin permeability coefficients (log Kp), total polar surface area (TPSA), number of rotatable bonds (nRotB), Gastrointestinal (GI) absorption, and CYP1A2 inhibitor were evaluated besides the Lipinski’s Rule of 5 (RO5), which predicts drug-likeness of the design derivatives were also considered. Lipinski’s RO5 states that compound in excesses of 5 H-bond donors, 10 H-bond acceptors, molecular weight more than 500 Da, and the calculated Log P (MLogP) greater than 5 likely had poor absorption or permeation of the molecular entities. Hence, molecules will unlikely to become orally bioavailable as a drug if they pose properties greater than the desired number (28).

## Results and Discussion

*QSAR model*

Several QSAR models were generated with a large value of the coefficient of determination; however, a model that is robust, efficient, and more reliable model was chosen as the best model based on the significance of its parameters since it has the largest value of R^2^ = 0.9465, R^2^_Adj _= 0.9304, Q^2^cv = of 0.8981, Q^2 ^_(L4O)_cv = 0.9272, and R^2^ext = 0.6915. The robustness and the predictive capacity of the QSAR model were predicted through the statistical parameters. The chosen model is presented below with the names, definitions, and coefficients of the descriptors listed in Table 2.

pEC_50 _= 5.79415(*ATSC5c*) - 9.38708(*MATS5e*) + 12.85927(*GATS8i*) - 10.11181(*SpMax2_Bhp*) + 18.90418(*PetitjeanNumber*) + 1.54996(*XLogP*) + 18.22399

N = 27, R^2 ^= 0.9465, R^2^_Adj_ = 0.9304, Q^2^cv = 0.8981, Q^2 ^_(L4O)_cv = 0.9272, LOF = 0.4280, R^2^_ext_ = 0.6915, N_ext_ = 7

*Model Validation*

**Figure 1:**
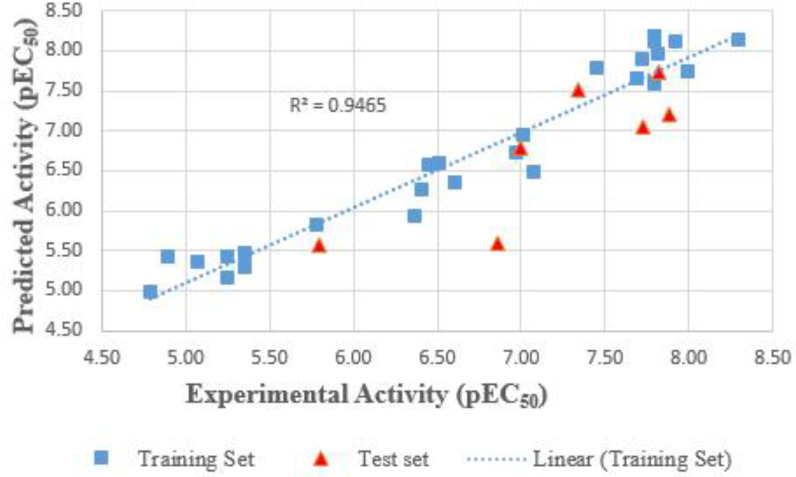
Experimental pEC_50_ plotted against predicted pEC_50_ for the dataset

The high value of Q^2^cv (0.8981), and that of Q^2 ^_(L4O)_cv = 0.9272 are indicators of good internal validations; the model was utilized externally to predict the activity of an external set which is reflected in the squared regression coefficient of the test set, R^2^_ext_ (0.6915). These results are a strong indication of the exclusive (internal and external) validation of a model. The plot of predicted activity against the experimental activity revealed a cluster of data points around the legend line, as shown in Figure 1, indicating the robustness and strength of the selected model. The small difference between the experimental and predicted activity (Table 1) emphasizes the accuracy of the model. Also, the Y-randomization test carried out shows the values of R^2^ and Q^2^ obtained after 15 repetitions are far smaller than their values in the model, confirming that the model does not occur by chance.

*Descriptors correlation matrix and Varia-nce inflation factor (VIF)*

The low variance in the correlation matrix (Table 3) between the model’s descriptors reveals a non-mutual relationship among the descriptors, which was supported by low values of calculated descriptors VIF (< 10) as found in Table 3. Indicating that the descriptors are found to be orthogonal (22), as such the model is statistically significant. 

*Applicability Domain (AD) of the model*

**Figure 2:**
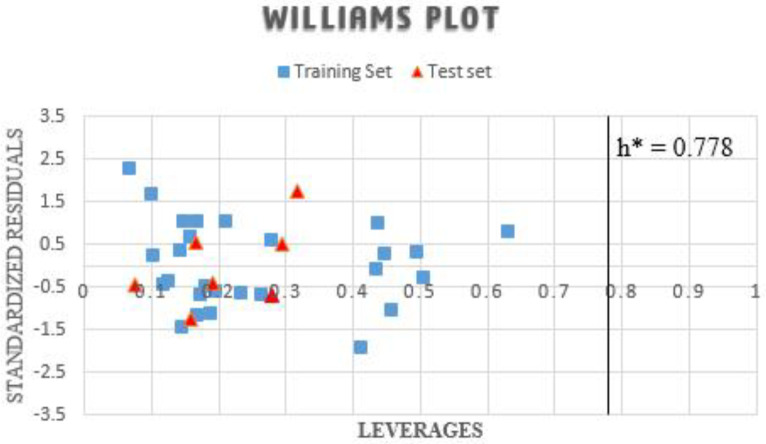
The plot of the standardized residuals against leverages

The model application limit defined by the applicability domain reflects the presents of the data sets within space, with no data point located outside the domain, as reflected in Figure 2. The threshold (h*) leverage is estimated for 0.778, beyond which the applicability of the models fails. Therefore, the whole dataset was found to possess decent leverage values and is within the model’s space, affirming the model’s predictive strength.

*Interpretation and contribution of descri-ptors*

The activity of the model, pEC_50 _= 5.79415(ATSC5c)-9.38708(MATS5e)+ 12.85927(GATS8i)- 10.11181 (SpMax2_Bhp) + 18.90418 (PetitjeanNumber) +1.54996(XLo-gP) +18.22399, is determined by the constituent descriptors ATSC5c, MATS5e, GATS8i, SpMax2_Bhp, PetitjeanNumber, and XLogP. The first descriptor, ATSC5c, which is defined as centered Broto–Moreau autocorrelation—lag 5/weighted by charges. The descriptor is related to the polarization of the molecules caused by highly electronegative elements present in a compound. The descriptor has a mean effect of MF = -0.3262 (Table 3) which indicates the activity increases with a decrease in the numeric values of the descriptors. The second descriptor, MATS5e belongs to the autocorrelation, and it describes the dependence of the compound on electronegativity (29). The autocorrelation descriptors check out the dependence of properties in one special molecule with the neighbor molecule and detect the conformity of the molecules (30). The mean effect (MF) analysis revealed the descriptor to have made MF = 0.0717 contribution. The positive sign of the MF indicates a positive contribution to the antimalarial activity. Hence, an increase in the value of the descriptor increases the antimalarial activity. The descriptor, GATS8i is a Geary autocorrelation of lag 8 weighted by first ionization potential. The 2D autocorrelation descriptors explained how the values of certain functions (topological distance) at intervals equal to the lag (atomic properties) were correlated. The analysis of the descriptors contribution yields the MF = -1.0598. The negative sign of the mean effect ensures the increase of activity with decrease descriptor values. SpMax2_Bhp is a Barysz matrix type descriptor in which the maximum absolute eigenvalue of Barysz matrix for n = 2 was weighted by polarizability (18). Analysis of the mean effect confirms SpMax2_Bhp to be the most contributive descriptor with MF = 3.3244, whose increase in numerical value increases the activity of compounds due to the positive MF. The value of shape parameter PetitjeanNumber increases when the substituents are changed from F, Cl to CF_3_, -OCH_3_ at a ring and hence increases the activity (31). The negative mean effect (MF = -0.7846) implies decreasing the descriptor values to increase the activity of the compound. The last descriptor, XlogP signifies the ratio of solute concentration in octanol & water and generally termed as octanol-water partition coefficient. The negative mean effect (MF = -0.2254) indicates decreasing the descriptor values to increase the compound activity. 

*Molecular design*

**Figure 3:**
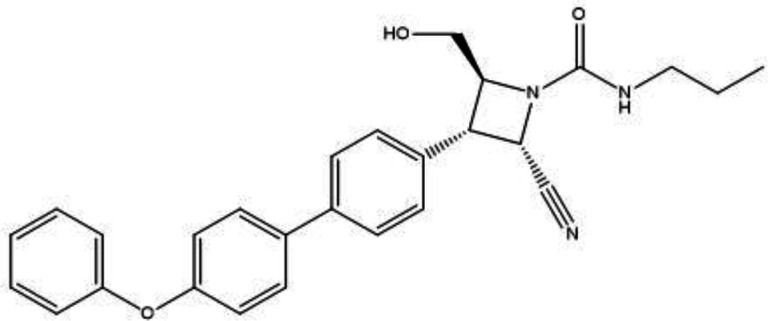
Design template, Compound 25, (2S,3S,4S)-2-cyano-4-(hydroxymethyl)-3-(4'-phenoxy-[1,1'-biphenyl]-4-yl)-N-propylazetidine-1-carboxamide, with pEC_50_ = 8.301

The compound with the highest activity (pEC_50_ = 8.301), compound 25 presented in Figure 3, was adopted as the design template. The descriptor, SpMax2_Bhp (a descriptor in which the maximum absolute eigenvalue of Barysz matrix for n = 2 was weighted by polarizability), was established as the most influential descriptor, was employed in the design of many speculative derivatives of Azetidine-2-carbonitriles. The descriptor relates to the polarizability of a molecule, and since it has a positive mean effect, increasing the polarizability of the compounds should be able to increase the antimalarial activity. Hence, polarizability can increase through the substitution of various electron deactivating groups (F, I, Cl, SO_3_H, CN, NO_2_, etc) at different positions of the template. This lead to the design of sixteen [16] speculative derivatives of the template as depicted in Table 4. Ten of the design derivatives (D3-4, D8-13, and D15-16) have better activity than the template. The compound D13 {(2S,3S,4S)-2-cyano-3-(2’-fluoro-4’-phenoxy-[1,1’-biphenyl]-4-yl)-4-(hydroxymethyl)-N-propylazetidine-1-carboxamide}, was found to have better antimalarial activity, (pEC_50_ = 9.8641) than those of the design template (pIC_50_ = 8.301), co-designed compounds as well as the chloroquine standard (pEC_50_ = 6.0242) as reflected in Table 4.

*Docking Protocol Validation*

The validation of the docking protocols was conducted to ascertain the docking method through the determination of the deviation of the re-docking output from the original docking pose. The deviation expressed as the root mean square deviation (RMSD) value produces the RMSD value of 1.895Å. This, therefore, validate the protocols employed in the docking and can be deployed in docking the designed ligands. 

*Docking Analysis*

**Figure 4:**
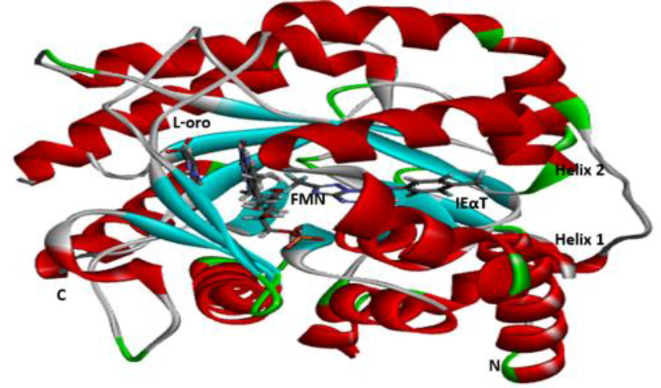
Ribbon diagram showing the indolyl-3-ethanone-α-thioethers binding site on PfDHODH. Indolyl-3-ethanone-α-thioethers is displayed as IEαT, FMN, and L-orotate

**Figure 5:**
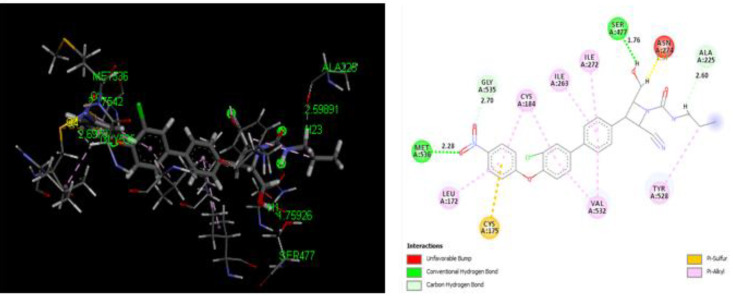
3- and 2-Dimensional docking pose of the interactions between D9 and the active site of the amino acid residues

The binding conformation of the design derivatives to the binding site of the target protein is discussed in the docking analysis. The structure of Plasmodium falciparum dihydroorotate dehydrogenase (Pf-DHODH) with the target site is reflected in Figure 4. Moreover, the docking result of the designed derivatives, template, and standard drug was shown in Table 5. The interactions of the ligand and the protein residues are analyzed, where hydrogen attached to either the hydroxyl or the Azetidine ring in most ligands showed H-bond interaction with Asp204 or Asp200 active site of the residues. The oxygen of the nitro in all the ligands shows H-bond interaction with either Lys305, Lys239, Lys559, Thr201, Ile206, Met536, Gly535, Asp216, or Asn195 active site residue, except in ligands D2, D3, D12, D13, D14, and D15. H-bond interaction could also be observed between the protein active site Lys239, Lys305, or Leu302 and Oxygen of N-propylacetamide of the ligands. Almost all compounds bar D1, D4, D11, D14, and D16, show H-bond interaction between the Asp200, Asp204, Ser202, Ser477, Ile218, Lys239, and Leu238 active site with methylene hydrogen of hydroxymethyl group of the compounds. Likewise, the oxygen of the hydroxyl group of the D2, D3, D14, and D15 ligands results in H-bond formation with Lys543, Lys239, Asn203, and Gly241 active sites of the protein residue. Seven of the designed derivatives, D2 (-150.8650 kcal/mol), D7 (-140.8770 kcal/mol), D9 (-177.0910 kcal/mol), D10 (-164.6990 kcal/mol), D12 (-150.2670 kcal/mol), D13 (-146.0110 kcal/mol), and D15 (-158.7300 kcal/mol), were found to possess higher binding affinity than the design template (-120.2690 kcal/mol) and the chloroquine standard (-140.3940 kcal/mol). Compound D9 was found to have the highest binding affinity (-177.0910 kcal/mol), as shown in Table 5. Hence, form better interaction than other designed derivatives as well as the standard chloroquine drug. Four H-bond in addition to several hydrophobic interactions were observed between D9 and the protein residue, two of which are conventional, between the oxygen of the nitro group of the ligand with Met536 protein residue, bond distance 2.28Å also, the interaction between the hydrogen of the methylene bridge bonded to a hydroxyl group of the ligand and Ser477 active site with bond distance 1.76Å. The other two interactions are carbon-hydrogen bonding between the oxygen of the ligand nitro group, hydrogen of N-propylacetamide with Gly535 bond distance 2.70Å, and Ala225, bond distance 2.60Å, respectively. Lastly, an unfavorable bump exists between the Asn274 residues with methylene hydrogen, which could add to the observer binding affinity. The binding modes for the best compound, D9, are presented in Figure 5. These interactions show the binding role of oxygen, hydrogen, and carbon atoms as well as their strength of inhibition.

*Drug-likeness ADME predictions*

The results of Lipinski’s parameters, drug-likeness as well as the *in-silico* ADMET screening predicted for the designed derivatives of Azetidine-2-carbonitriles were depicted in Table 6. The results show that all the designed derivatives obeyed Lipinski’s rule of five, hence possess excellent drug-like properties (32), other parameters like molar refractivity (MR), and the number of rotatable bonds (nRotB) were determined in addition to Lipinski’s parameters. Molar refractivity measures both the ease of polarization and volume of a compound; it ranges between 40 -130 (33). The rule is deployed to assess the drug-likeness of a drug candidate (34). The nRotB measures the molecular flexibility of the molecule, which should be ≤ 10. The violation of more than one rule of five by a drug candidate is a pointer to the poor oral absorption of the candidate. The great combination of membrane permeability and oral bioavailability are functions of the Log of octanol/water partition coefficient (LogP), Molecular weight (MW), and Total polar surface area (TPSA) values. In addition to the role played by hydrogen bond acceptor (HBA) and hydrogen bond donor (HBD) in determining the hydrophobicity, membrane permeability, and the bioavailability of drug candidates. The results in Table 6 indicate that all compounds are within the parameter range of MW ≤ 500 Da, LogP < 5, nHBD ≤ 5, nHBA ≤ 10, and TPSA < 140 Å^2^. This shows that the design derivatives are not only bioavailable, they are also membrane-permeable besides their hydrophobicity nature. The predicted ADME values (Table 6) have the skin permeability (log Kp) for the design compounds to be within -6.31 to -5.69 cm/s, lying between the acceptance range –8.0 to –1.0 cm/s (35). With the values of the nRotB ≤10, those of the MR were slightly outside the range. While most compounds showed low gastrointestinal absorption with only compounds D1-3, D13-15 that have high absorption, only a few compounds, D1, D2, D3, D14, and D15, show inhibition to CYP1A2. 

**Table 1:** Chemical structures and activities of the derivatives of Azetidine-2-carbonitriles against Chloroquine resistance strain, Dd2

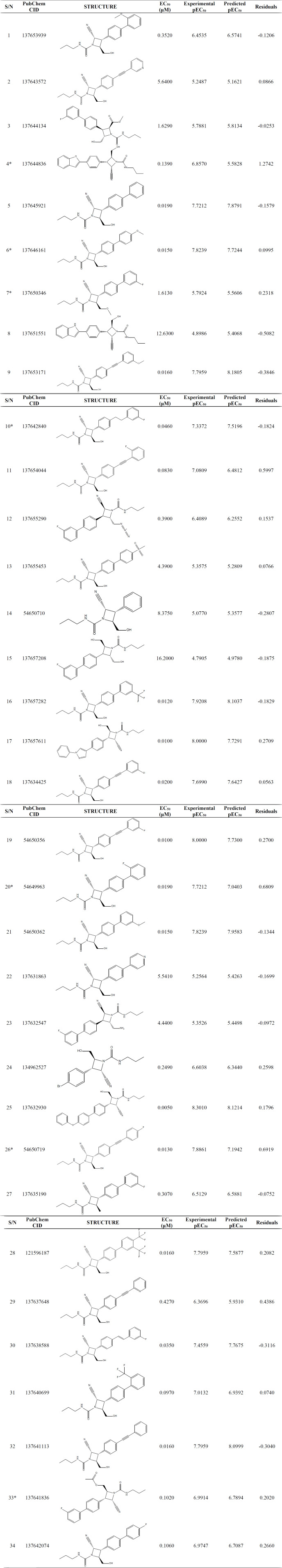

**Table 2:** Names, definitions, and coefficients of descriptors appearing in the selected model

	**Descriptor name**	**Type**	**Notation**	**Coefficient**
			Constant	18.22
1	Centered Broto-Moreau autocorrelation - lag 5/weighted by charges	2D-Autocorrelation	ATSC5c	5.79
2	Moran autocorrelation - lag 5/weighted by Sanderson electronegativities	2D-Autocorrelation	MATS5e	-9.39
3	Geary autocorrelation - lag 8/weighted by first ionization potential	2D-Autocorrelation	GATS8i	12.86
4	Largest absolute eigenvalue of Barysz matrix - n 2 / weighted by relative polarizabilities	Barysz matrix	SpMax2_Bhp	-10.11
5	Petitjean number	Petitjean number	PetitjeanNumber	18.90
6	XLogP	XLogP	XLogP	1.55

**Table 3:** Descriptors correlation matrix, VIF, and their Mean effect

	**pEC**_50_	**ATSC5c**	**MATS5e**	**GATS8i**	**SpMax2_Bhp**	**Petitjean** **Number**	**XLogP**	**VIF**	**MF**
pEC_50_	1								
ATSC5c	0.0516	1						2.3640	-0.3262
MATS5e	0.0729	0.5890	1					3.0033	0.0717
GATS8i	0.2138	-0.1170	0.3532	1				2.6423	-1.0598
SpMax2_Bhp	0.2163	-0.0471	-0.1380	0.2733	1			1.8832	3.3244
Petitjean Number	0.3992	0.0425	0.0150	0.2741	0.1633	1		1.1472	-0.7846
XLogP	0.7071	-0.0473	-0.0205	-0.2401	0.3923	-0.0038	1	1.7121	-0.2254

**Table 4:** Structures of the template, designed derivatives of Azetidine-2-carbonitriles and Chloroquine standard along with their respective activities

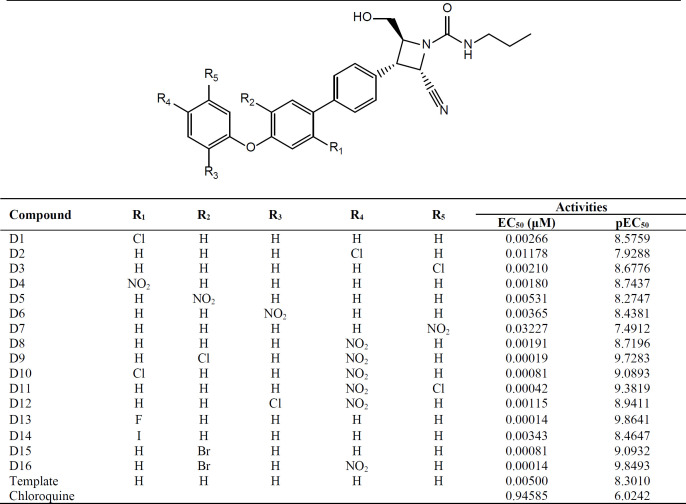

**Table 5:** Docking parameters of designed derivatives of Azetidine-2-carbonitriles, template, and chloroquine standard in the active site of PfDHODH protein

**Compound No.**	**MolDock Score ** **(kcal/mol)**	**No. of H-Bonds**	**Amino acid involved**	**Atom of ligands**	**H-bond length (Å)**
D1	-128.8790	2	Lys305	O of NO_2_	2.48
			Asp204	H of OH	2.14
D2	-150.8650	11	Lys543	O of OH	2.75
			Lys543	O of OH	2.76
			Ser202	H of OH	2.14
			Leu302	H of Amide	2.49
			Lys239	O of N-propylacetamide	2.71
			Leu302	N of CN	2.65
			Asp200	H of CH_2_ of hydroxyl methyl	3.00
			Ser202	H of CH_2_ of hydroxyl methyl	2.64
			H	O	2.41
			Asp204	H of Azetidine ring	2.83
			Leu302	H of N-propylacetamide	2.89
D3	-128.8700	3	Asp200	H of OH	1.74
			Lys239	O of OH	2.84
			Asp200	H of the CH_2_ of hydroxymethyl	2.54
D4	-133.4450	3	Lys305	O of N-propylacetamide	2.42
			Asp204	H of OH	2.13
			Thr201	O of NO_2_	2.83
D5	-122.6040	5	Lys305	O of N-propylacetamide	2.33
			Asp204	H of OH	2.17
			Thr201	O of NO_2_	2.46
			Thr201	O of NO_2_	3.08
			Asp204	H of the CH_2_ of hydroxymethyl	2.79
D6	-139.4120	4	Leu238	H of OH	2.46
			Ile206	O of NO_2_	2.67
			Asp200	H of the CH_2_ of hydroxymethyl	2.10
			Asp200	H of Azetidine ring	2.55
D7	-140.8770	3	Leu238	H of OH	2.38
			Asp200	H of the CH_2_ of hydroxymethyl	2.20
			Asp200	H of Azetidine ring	2.32
D8	-124.5920	6	Lys239	O of NO_2_	2.46
			Lys305	O of N-propylacetamide	2.69
			Lys305	O of N-propylacetamide	2.55
			Asp204	H of OH	1.64
			H	O of OH	3.09
			Ile218	H of the CH_2_ of hydroxymethyl	2.96
D9	-177.0910	4	Met536	O of NO_2_	2.28
			Ser477	H of the CH_2_ of hydroxymethyl	1.76
			Gly535	O of NO_2_	2.70
			Ala225	H of N-propylacetamide	2.60
D10	-164.6990	7	Lys559	O of NO_2_	2.36
			Leu238	H of OH	2.10
			Asp200	H of Amide	2.02
			Asp216	O of NO_2_	2.90
			Asp200	H of the CH_2_ of hydroxylmethyl	2.56
			Lys239	H of the CH_2_ of hydroxylmethyl	2.96
			Asp200	H of Azetidine ring	2.38
D11	-125.9140	4	Asn195	O of NO_2_	2.44
			Lys239	O of NO_2_	1.97
			Lys305	O of N-propylacetamide	2.35
			Asp204	H of OH	2.20
D12	-150.2670	6	Lys305	O of Oxydibenzene	2.71
			Lys239	H of OH	2.14
			Asp200	H of Amide	2.08
			Leu238	H of the CH_2_ of hydroxymethyl	2.88
			Asp200	H of Azetidine ring	2.10
			H	O of OH	2.80
D13	-146.0110	4	Asp200	H of OH	1.75
			Leu302	N of CN	2.73
			Ser202	H of the CH_2_ of hydroxymethyl	2.25
			Asp204	H of Azetidine ring	2.90
D14	-137.2260	7	Thr201	H of OH	1.97
			His306	H of Amide	2.59
			Asn203	O of OH	2.91
			H of CH_2_ of hydroxymethyl	O of N-propylacetamide	2.62
			Ser202	H of Azetidine ring	2.88
			Leu302	O of N-propylacetamide	2.85
			Asp204	H of a delocalized benzene ring	2.99
D15	-158.7300	6	Leu238	H of OH	2.09
			Asp200	H of Amide	2.14
			Gly241	O of OH	2.43
			Asp200	H of the CH_2_ of hydroxymethyl	2.45
			Lys239	H of the CH_2_ of hydroxymethyl	2.89
			Asp200	H of Azetidine ring	2.42
D16	-134.8030	3	Lys239	O of NO_2_	1.89
			Lys305	O of N-propylacetamide	2.48
			Asp204	H of OH	2.13
Template	-120.2690	3	Lys305	O of N-propylacetamide	2.56
			Asp204	H of OH	2.17
			Asp204	H of the CH_2_ of hydroxymethyl	2.97
Chloroquine	-140.3940	2	His185	N of Quinoline ring	1.54
			Val532	H of amine	2.67

**Table 6:** Lipinski properties of the derivatives of Azetidine-2-carbonitriles analyzed with SwissADME

	**Lipinski’s parameters**						**MR**	**log Kp (cm/s)**	**nRotB** **(≤10)**	**GI absorption**	**CYP1A2 inhibitor**
**S/N**	**MW ** **(≤500 Da)**	**MLogP (<5)**	**nHBD (≤5)**	**nHBA (≤10)**	**TPSA (<140 Å2)**	**Lipinski Violation**					
D1	475.97	3.42	2	4	85.59	0	135.82	-5.69	9	High	Yes
D2	475.97	3.42	2	4	85.59	0	135.82	-5.69	9	High	Yes
D3	475.97	3.42	2	4	85.59	0	135.82	-5.69	9	High	Yes
D4	486.52	2.07	2	6	131.41	0	139.64	-6.31	10	Low	No
D5	486.52	2.07	2	6	131.41	0	139.64	-6.31	10	Low	No
D6	486.52	2.07	2	6	131.41	0	139.64	-6.31	10	Low	No
D7	486.52	2.07	2	6	131.41	0	139.64	-6.31	10	Low	No
D8	486.52	2.07	2	6	131.41	0	139.64	-6.31	10	Low	No
D9	520.96	2.53	2	6	131.41	1	144.65	-6.08	10	Low	No
D10	520.96	2.53	2	6	131.41	1	144.65	-6.08	10	Low	No
D11	520.96	2.53	2	6	131.41	1	144.65	-6.08	10	Low	No
D12	520.96	2.53	2	6	131.41	1	144.65	-6.08	10	Low	No
D13	459.51	3.32	2	5	85.59	0	130.77	-5.96	9	High	No
D14	567.42	3.61	2	4	85.59	1	143.53	-6.23	9	High	Yes
D15	520.42	3.51	2	4	85.59	1	138.51	-5.91	9	High	Yes
D16	565.42	2.63	2	6	131.41	1	147.34	-6.31	10	Low	No

MW: Molecular weight; LogP: Log of octanol/water partition coefficient;GI (Gastrointestinal) absorption; nHBA: Number of hydrogen bond acceptor(s); nHBD: Number of hydrogen bond donor(s), CYP1A2: Cytochrome P450 family 1 subfamily A member 2, MR-Molar refractivity, nRotB: Number of rotatable bonds; TPSA: Total polar surface area; log Kp: Log of skin permeability.

## Conclusion

In this research, the ligand-base method was adopted to design sixteen (16) derivatives of Azetidine-2-carbonitriles. Ten of the designed derivatives (D3-4, D8-13, and D15-16) have better activity than the template. Molecular docking studies of the derivatives show the various interactions and the binding sites of the compounds. The compound with the highest binding energy (D9) shows its interaction with Met536, Ser477, Gly535, and Asn274 amino acid residues, which may be responsible for the high antimalarial activities. The designed compounds were found to pass all Lipinski’s RO5. The compounds were found to have their skin permeability coefficient within limits, with most of them having low gastrointestinal absorption, while compounds D1, D2, D3, D14, and D15 show inhibition to CYP1A2. 

## Authors’ contributions

This research involves the combined efforts of all the authors. ZY. Ibrahim and A. Uzairu, Conceived and designed the research, ZY. Ibrahim and G. Shallangwa, Performed the experiments, ZY. Ibrahim, A. Uzairu, and S. Abechi Analyzed and interpreted the data, ZY. Ibrahim, G. Shallangwa, and S. Abechi Contributed materials, analysis tools, or data, ZY. Ibrahim and S. Abechi, Wrote the manuscript. All authors read and approved the final manuscript.

## Funding

The authors of this research did not receive any funding concerning this research.

## Availability of data

The datasets used for analysis during these studies were included in this published study.

## Ethics approval and consent to participate

This research does not require ethical approval.

## Consent for publication

On behave of the authors, I hereby granted the right of this entire article content to this journal.

## Declaration of Conflicting Interests

The author(s) declared no potential conflicts of interest concerning the research, authorship, and/or publication of this article.
